# Disease‐Intrinsic Organising Pneumonia During Biomarker‐Confirmed Ulcerative Colitis Remission

**DOI:** 10.1002/rcr2.70702

**Published:** 2026-09-08

**Authors:** Rahaf A. Jereisat, Nawras Ibrahim, Maher Dahdel

**Affiliations:** ^1^ Department of Internal Medicine University of Houston/HCA‐Clear Lake Houston Texas USA

**Keywords:** cryobiopsy, extraintestinal manifestation, organising pneumonia, ulcerative colitis

## Abstract

Organising pneumonia is an increasingly recognised pulmonary manifestation of ulcerative colitis, though nearly all reported cases are attributed to drug‐induced aetiology. The 2025 ERS/ATS classification of interstitial pneumonias now explicitly lists ulcerative colitis among secondary causes of cicatricial organising pneumonia, formally recognising a disease‐intrinsic mechanism for organising pneumonia in ulcerative colitis. We present a 76‐year‐old woman with 10‐year ulcerative colitis pancolitis on stable mesalamine in biochemical remission (faecal calprotectin 62 μg/g, below the 80 μg/g histological activity threshold) who developed progressive dyspnea. Drug‐induced aetiology was systematically excluded: mesalamine had been stable for 10 years, discontinuation produced no improvement, and corticosteroids were required for response. Transbronchial cryobiopsy was initially interpreted as fibrotic non‐specific interstitial pneumonia; expert pathology consultation revised the diagnosis to organising pneumonia with Masson bodies, changing management from antifibrotics to corticosteroids, illustrating the clinical consequences of cryobiopsy diagnostic discordance. Bronchoalveolar lavage revealed a neutrophil‐predominant profile (42% neutrophils, 3% lymphocytes), atypical for cryptogenic organising pneumonia, which typically shows lymphocytic predominance. This pattern may reflect gut‐lung axis immune trafficking in secondary organising pneumonia associated with ulcerative colitis. Concurrent seronegative arthritis was reclassified as ulcerative colitis‐associated spondyloarthropathy, removing rheumatoid arthritis‐associated interstitial lung disease from the differential. This case extends the sole prior report of organising pneumonia during confirmed ulcerative colitis remission by incorporating biomarker‐confirmed remission, systematic drug exclusion, cryobiopsy discordance with treatment‐altering consequences and contextualisation within the updated 2025 ERS/ATS classification framework.

## Graphical abstract

Organising pneumonia (OP) can occur as a disease‐intrinsic extraintestinal manifestation of ulcerative colitis (UC) during biochemical remission. Drug‐induced aetiology must be systematically excluded before attributing OP to UC. Cryobiopsy‐based ILD diagnoses require expert pathology review when treatment decisions are consequential, as the distinction between intraluminal Masson bodies and interstitial fibroblast foci directly determines management. A neutrophil‐predominant BAL profile in IBD‐associated OP may reflect gut‐lung axis immune trafficking and warrant investigation in future cases.

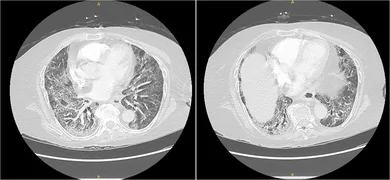

## 1 Introduction

Ulcerative colitis (UC) is associated with extraintestinal manifestations (EIMs) in up to 27% of patients [1]. Pulmonary manifestations are increasingly recognised: Moda et al. identified lung disease in 5.0% of 563 UC patients, with organising pneumonia (OP) in 1.8% [2]. Notably, 9 of 10 OP cases in that cohort were attributed to drug‐induced pathogenesis, leaving the question of whether OP can occur as a primary, disease‐intrinsic EIM largely unanswered.

The 2025 ERS/ATS update to the international multidisciplinary classification of interstitial pneumonias now explicitly lists UC among secondary causes of cicatricial OP (CiOP), a histologically distinct entity, thereby providing formal recognition that OP can occur as a disease‐intrinsic manifestation of UC [3].

Only one prior case was described of OP occurring after histologically confirmed UC remission [4]. Here, we present a case that extends that observation through biomarker‐confirmed remission, systematic exclusion of drug‐induced aetiology, cryobiopsy diagnostic discordance with treatment‐altering consequences and an atypical bronchoalveolar lavage (BAL) profile with implications for the gut‐lung axis.

## 2 Case Report

A 76‐year‐old never‐smoking woman with a 10‐year history of UC pancolitis on stable mesalamine 2.4 g daily in clinical remission presented with progressive dyspnea over 1 month. She carried a concurrent diagnosis of rheumatoid arthritis (RA), made 5 years after UC onset based on bilateral symmetric polyarthritis involving 15 small joints with finger nodules. Adalimumab (2 years) and certolizumab (1 year) had been sequentially trialled and discontinued for lack of efficacy. She had a history of deep vein thrombosis on apixaban. Pulmonary function tests 3 months prior were normal.

On admission, oxygen saturation was 99% on 4 L/min nasal cannula with bilateral inspiratory crackles. Faecal calprotectin obtained 1 month prior was 62 μg/g, below the 80 μg/g cutoff identifying histological activity, consistent with biochemical remission [5]. Inflammatory markers were elevated: CRP 24.1 mg/L, ESR 63 mm/h, WBC 19.0 × 10^3^/μL with 81.8% neutrophils. Creatine kinase was normal (44 U/L). Autoimmune serology showed RF negative, anti‐CCP negative (specificity > 95% for RA), ANA 1:160 with monospecific dense fine speckled (DFS70) pattern (found in fewer than 1% of established systemic autoimmune rheumatic diseases), HLA‐B27 negative and all other autoantibodies negative (anti‐dsDNA, anti‐RNP, anti‐Smith, C‐ANCA, P‐ANCA, atypical pANCA, PR3, MPO) [6, 7]. A triple‐phase bone scan demonstrated sacroiliac joint uptake.

**FIGURE 1:**
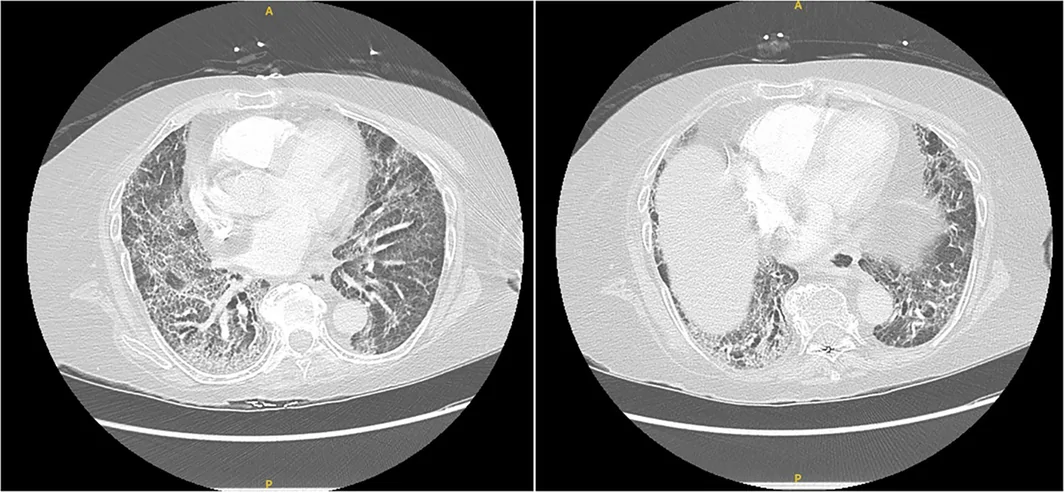
Axial HRCT of the chest at presentation. This Figure shows diffuse bilateral interstitial haziness predominantly in the mid and lower lung fields. Interlobular septal thickening is present without focal consolidation, honeycombing or pleural effusion.

CT pulmonary angiography excluded pulmonary embolism. High‐resolution CT (HRCT) demonstrated bilateral interstitial haziness predominantly in the mid and lower lung fields (Figure 1), representing progression from prior imaging that had shown only mild scattered scarring.

Transbronchial cryobiopsy of the right lower lobe was performed. BAL differential showed 42% neutrophils, 3% lymphocytes, 2% monocytes and 53% lining cells. Initial histopathological interpretation reported fibrotic non‐specific interstitial pneumonia (NSIP), and mycophenolate plus nintedanib was planned.

**FIGURE 2:**
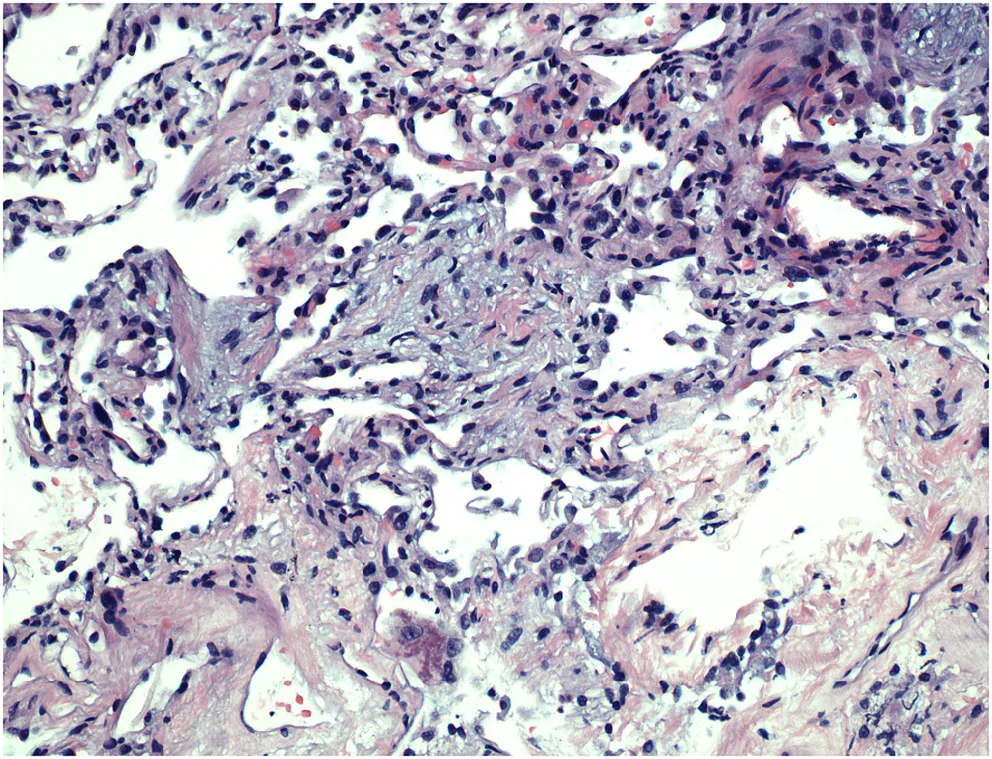
Organising pneumonia with Masson bodies (H&E). Transbronchial cryobiopsy showing an intraluminal polypoid plug of loose fibroblastic connective tissue (Masson body) within an alveolar space, with preserved underlying architecture and absence of interstitial fibrosis. Initial interpretation was fibrotic NSIP; expert consultation revised the diagnosis to organising pneumonia, a recognised pulmonary manifestation of ulcerative colitis, changing treatment from antifibrotics to corticosteroids.

Expert pathology consultation (Cleveland Clinic) revised the interpretation: ‘Sections show organizing pneumonia, characterised by the presence of a few polypoid fibroblast plugs (Masson bodies) within airspaces. There is mild fibrosis in the background, but significant scarring, honeycomb change or fibroblast foci are absent. Features of named forms of interstitial lung disease are absent’ (Figure 2).

Mesalamine was held as a precautionary measure. The patient received pulse methylprednisolone 500 mg IV daily for 3 days, followed by oral prednisone 0.5 mg/kg with a planned 6‐month taper and Pneumocystis jirovecii prophylaxis. Follow‐up CT at 2 months showed partial resolution of bilateral infiltrates with residual subpleural fibrosis and traction bronchiectasis in the lower lobes, similar to pre‐existing baseline changes. Prednisone was increased to 30 mg daily, and azathioprine was added as a steroid‐sparing agent with dual utility for UC maintenance.

## 3 Discussion

Three observations argue against drug‐induced aetiology. First, mesalamine exposure had been stable for 10 years; drug‐induced pneumonitis typically presents within weeks to months of initiation or dose change [2]. Second, mesalamine was held during hospitalisation without clinical improvement, and corticosteroids were required for response; inconsistent with a drug reaction expected to improve on discontinuation. Third, the 2025 ERS/ATS classification now explicitly lists UC among secondary causes of cicatricial OP; a histologically distinct entity characterised by dense fibrous bands and dendriform ossification, thereby formally recognising a disease‐intrinsic mechanism for OP in UC, independent of drug exposure [3].

The occurrence of OP during biochemical remission is consistent with the established behaviour of pulmonary EIMs in UC. Camus et al. documented that respiratory involvement can arise independently of bowel disease activity, including after colectomy, in 8 of 28 cases with available temporal data [8]. This case extends the sole prior report by Jang et al. in several dimensions (Table 1) [4]. Remission was confirmed by a validated biomarker (FC 62 μg/g, below the 80 μg/g histological activity cutoff) rather than colonoscopic biopsy alone [5]. The drug‐induced versus disease‐intrinsic question was explicitly addressed. While two cases over 13 years may reflect genuine rarity, under‐recognition is equally plausible given that drug‐induced aetiology was attributed to 9 of 10 OP cases in the Moda et al. cohort without systematic exclusion criteria [2].

**TABLE 1:** Comparison with the only prior published case of organising pneumonia in UC remission.

Feature	Jang et al. [4]	Present case
Remission confirmation	Histological (colonoscopic biopsy)	Biochemical: FC 62 μg/g; below 80 μg/g histological activity cutoff [5]
Drug‐induced versus disease‐intrinsic	Not explicitly addressed	Systematically excluded: 10‐year stable mesalamine; no improvement after discontinuation; 2025 ERS/ATS classification lists UC among secondary causes of CiOP, supporting disease‐intrinsic mechanism [2, 3]
BAL data	Not reported	42% neutrophils, 3% lymphocytes; atypical for COP [9]
Pathological method	Not specified	Transbronchial cryobiopsy with expert consultation discordance (NSIP → OP) [10, 11]
Autoimmune workup	Not reported	Full panel (RF, anti‐CCP, HLA‐B27, DFS70, ANCA); excluded RA; reclassified to UC‐SpA [6, 7]
Guideline context	Pre‐2025 classification	2025 ERS/ATS lists UC as a secondary cause of CiOP, formally recognising disease‐intrinsic UC–OP [3]

The discordance between local (fibrotic NSIP) and expert (OP with Masson bodies) interpretations reflects a recognised cryobiopsy pitfall. Churg et al. published the first systematic guide to interpreting cryobiopsies for diffuse parenchymal lung disease and identified the key discriminating feature: intraluminal polypoid plugs (Masson bodies) clearly situated within airspaces favour OP, whereas interstitial fibroblast foci (located within the alveolar wall) characterise UIP/NSIP [10]. The CAN‐ICE trial documented only fair between‐center agreement (κ = 0.29) for cryobiopsy‐based diagnoses [11]. In this case, the discordance was directly consequential: The planned antifibrotic regimen was replaced by corticosteroid monotherapy, which carries over 90% five‐year survival in OP [9]. Expert pathology review should be considered routine when cryobiopsy‐based ILD diagnoses drive major treatment decisions.

The BAL profile (42% neutrophils, 3% lymphocytes) is atypical for classic cryptogenic OP, which typically shows lymphocytic predominance with milder increases in neutrophils and eosinophils [9]. Choi et al. found that BAL lymphocyte counts were significantly higher in cryptogenic than in secondary OP (*p* = 0.012), suggesting secondary OP may carry a different cellular signature [12]. We hypothesise that UC‐associated secondary OP may exhibit a neutrophil‐predominant BAL reflecting gut‐lung axis immune cell trafficking; supported by murine data demonstrating colitis‐induced pulmonary neutrophil infiltration via IL‐17 elevation and microbial translocation [13], IL‐6‐driven neutrophil‐mediated pulmonary inflammation associated with bacteremia [14] and neutrophil expansion in colitis‐associated lung inflammation [15]. This hypothesis requires validation in future cases (Table 2).

**TABLE 2:** BAL differential comparison table.

Diagnosis	Lymphocytes	Neutrophils	Eosinophils	Key BAL feature
This patient (UC‐associated OP)	3%	42%	Not reported	Markedly neutrophil‐predominant; atypical for classic OP
Cryptogenic OP (COP)	20%–40% (↑↑)	5%–10% (mild ↑)	2%–5% (mild ↑)	Lymphocyte‐predominant with mild increases in all cell types
Secondary OP	Lower than COP	Higher than COP	Variable	Lower lymphocytes and higher neutrophils compared to COP
Fibrotic NSIP	20%–50% (↑↑)	Mildly ↑	Variable	Lymphocyte‐predominant; similar to COP
UIP/IPF	Normal–mild ↑	5%–15% (↑)	Variable	Neutrophil‐predominant; lymphocytes typically not elevated

*Note:* This table compares the patient's BAL cellular profile against published profiles for COP, secondary OP, fibrotic NSIP and UIP, highlighting the atypical neutrophil‐predominant pattern observed in this case.

Seronegative status (anti‐CCP specificity > 95% for RA), monospecific DFS70 ANA pattern (found in < 1% of established systemic autoimmune rheumatic diseases) [6, 7], negative HLA‐B27, sacroiliac joint uptake and onset 5 years after UC collectively argue against RA and favour UC‐associated spondyloarthropathy; comprising Type 2 peripheral arthritis (polyarticular, symmetric, potentially independent of bowel activity) and axial involvement [1]. This reclassification removes RA‐associated ILD from the differential and identifies UC as the sole autoimmune driver, ensuring management is directed at IBD‐related pathology.

Pulmonary function tests were not obtained during hospitalisation. Surgical lung biopsy was not performed. Long‐term follow‐up data are not yet available. The BAL hypothesis is based on a single case with murine supporting data.

OP can occur as a disease‐intrinsic extraintestinal manifestation of UC during biochemical remission. The 2025 ERS/ATS classification supports this by listing UC among secondary causes of cicatricial OP, formally recognising a disease‐intrinsic mechanism for OP in UC. Drug‐induced aetiology must be systematically excluded before attributing OP to UC. Cryobiopsy‐based ILD diagnoses require expert pathology review when treatment decisions are consequential, as the distinction between intraluminal Masson bodies and interstitial fibroblast foci directly determines management. A neutrophil‐predominant BAL profile in IBD‐associated OP may reflect gut‐lung axis immune trafficking and warrants investigation in future cases.

## Consent

The authors declare that written informed consent was obtained for the publication of this manuscript and accompanying images and attest that the form used to obtain consent from the patient(s) complies with the Journal requirements as outlined in the author guidelines.

## Conflicts of Interest

The authors declare no conflicts of interest.

## Data Availability

The data that support the findings of this study are available on request from the corresponding author. The data are not publicly available due to privacy or ethical restrictions.
